# Theoretical Analysis and Determination of the Correction Factor for a Waveguide Microcalorimeter

**DOI:** 10.3390/s20010245

**Published:** 2019-12-31

**Authors:** Xiaohai Cui, Yu Song Meng, Wenze Yuan, Yong Li

**Affiliations:** 1 National Institute of Metrology, Beijing 100013, China; yuanwz@nim.ac.cn (W.Y.); liy@nim.ac.cn (Y.L.); 2 National Metrology Centre, Agency for Science, Technology and Research (A*STAR), Singapore 118221, Singapore; meng_yusong@nmc.a-star.edu.sg

**Keywords:** correction factor, electromagnetic field theory, microcalorimeter, primary standards

## Abstract

This paper proposes a new method for determining the correction factor of a newly developed waveguide primary power measurement system (i.e., microcalorimeter), based on the electromagnetic field theory analysis for waveguide thermal isolation section (TIS) in foil short measurement mode. The new method determines the contribution of the power dissipated within the TIS into the correction factor, in term of the physical dimensions of the TIS. Performance comparison and analysis show that the newly proposed method can significantly reduce the measurement uncertainty when evaluating the correction factor of waveguide microcalorimeters.

## 1. Introduction

Microcalorimeters have been recognized to be an effective solution for radio frequency (RF), microwave, and millimeter-wave power measurements [1,2,3], and have been successfully developed within the National Metrology Institutes worldwide over past few decades [4,5,6,7,8]. The main function and application of a microcalorimeter is to determine the effective efficiency $\eta_{e}$ of a transfer standard (e.g., a thermistor mount, and referred as a device under test (DUT) in this paper), and its correction factor g is found to be critical and has been studied in many different ways [9,10,11,12].

In the millimeter-wave range, waveguide microcalorimeter has been adopted due to its good reliability and accuracy up to 110 GHz [8,10,11,12] or further. To accurately determine its correction factor g, a method based on the measurement of offset shorts of different length followed by one single calibration measurement of a DUT has been proposed in [10]. Recently, another method based on attaching a thermistor sensor into the waveguide thermal isolation section (TIS) to accurately measure its temperature change has been proposed in [12]. Both the methods are found to have good performance during the evaluations of a WR-22 (33–50 GHz) waveguide microcalorimeter.

However, as the frequency of interest further increases, the size of waveguide becomes smaller which motivates us to find other solutions without using extra fixtures/accessories. As a continued work of [13], theoretical analysis and modeling of the correction factor g of a waveguide microcalorimeter will be performed in this paper, in terms of the physical dimensions of its TIS, based on the electromagnetic field theory analysis. The proposed solution tends to eliminate the usage of external fixtures, and reduce the measurement uncertainty when calibrating a RF/microwave/millimeter-wave power sensor with waveguide connection. For simplicity in the rest of this paper, RF will be synonymous for RF, microwave, and millimeter-wave.

In the remainder of this paper, the theoretical background and operation principle of a waveguide microcalorimeter is discussed in Section 2. This is followed by the proposal of a new method for determining its correction factor g in Section 3. In Section 4, detailed description of electromagnetic field theory analysis for waveguide TIS in foil short measurement mode will be carried out. Performance comparison of the new method will be given in Section 5. Finally, conclusion of this paper will be drawn in Section 6.

## 2. Theoretical Background and Operation Principle

The effective efficiency $\eta_{e}$ of a thermistor mount (a type of power sensor which is widely used for precision RF power measurements) is important for accurate determination of total power $P_{rf}$ dissipated within the thermistor mount, and is defined as

(1) $$\eta_{e}=\frac{P_{sub}}{P_{rf}}.$$

where $P_{sub}$ is the direct current (DC) substituted power of the thermistor mount and calculated in term of its bias voltages without and with RF signal applied (i.e., *V*_1_ and *V*_2_) at a steady status as follows,

(2) $$P_{sub}=\frac{V_{1}^{2}-V_{2}^{2}}{R}.$$

**Figure 1:**
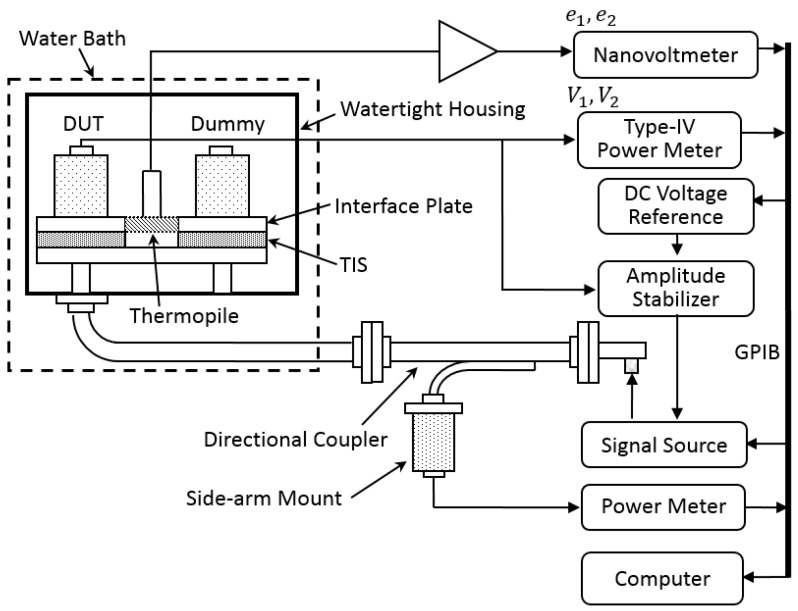
Schematic diagram for the setup and operation of a waveguide microcalorimeter.

Here, *R* is the operating resistance of the thermistor mount. Figure 1 below shows a detailed measurement setup for determining the effective efficiency $\eta_{e}$ of a DUT thermistor mount. Its core part consists of a thermally insulated microcalorimeter (twin-line structure) including a thermopile and a thermal reference (dummy), and a Type-IV power meter. *V*_1_ and *V*_2_ are measured by the Type-IV meter directly.

According to the law of conservation of energy, the unsubstituted portion (labeled as $P_{w}$, and $P_{rf}=P_{sub}+P_{w}$) of total dissipated power $P_{rf}$ can cause relative temperature rise of the DUT mount referring to the Dummy mount, which is monitored by the thermopile as shown in Figure 1, and supposed to be indicated by thermopile output voltage change $\Delta e=e_{2}-e_{1}$, where *e*_1_ and *e*_2_ are the output voltages of the thermopile corresponding to *V*_1_ and *V*_2_ and measured by a nanovoltmeter. It is noted that this unsubstituted power $P_{w}$ is the portion of RF power dissipated but does not affect the reading *V*_2_ of the nanovoltmeter.

### 2.1. Definition of the Correction Factor

However, the power $P_{i}$ dissipated at its waveguide TIS can also contribute to Δe, and therefore has to be differentiated. Without differentiation, the output voltage change Δe of thermopile includes the contribution from $P_{w}$ and $P_{i}$, with the following relationship,

(3) $$\Delta e=k\left(P_{w}+cP_{i}\right),$$

where k is a proportionality constant that depends on the fraction of power that is detectable by the thermopile and the thermopile sensitivity [11], and c is an equivalence factor that considers the thermal paths which are different comparing to those from the mount to the thermopile.

The uncorrected effective efficiency $\eta_{e,uncor}$ [7] comparing to the effective efficiency $\eta_{e}$ in (1), including the contribution from $P_{i}$ to the thermopile output voltage change Δe, and is defined as

(4) $$\eta_{e,uncor}=\frac{P_{sub}}{P_{rf}+cP_{i}}=\frac{P_{sub}}{P_{sub}+P_{w}+cP_{i}}=\frac{P_{sub}}{P_{sub}+\frac{\Delta e}{k}}.$$

The correction factor g of a microcalorimeter is then defined as,

(5) $$g=\frac{\eta_{e}}{\eta_{e,\;uncor}}=1+\frac{cP_{i}}{P_{rf}}.$$

The correction factor g is used to remove the contribution of $P_{i}$ from the directly calculated uncorrected effective efficiency $\eta_{e,uncor}=\left[1-{\left(\frac{V_{2}}{V_{1}}\right)}^{2}\right]/\left[\frac{e_{2}}{e_{1}}-{\left(\frac{V_{2}}{V_{1}}\right)}^{2}\right]$ [7,12] with the measured *V*_1_, *V*_2_, *e*_1_, and *e*_2_ using the hardware setup in Figure 1.

### 2.2. System Constant

For a thermistor mount with input reflection coefficient of $\Gamma_{M}$ and incident power of $P_{IM}$, the dissipated power $P_{i}$ at the TIS due to both the forward and reverse transmissions is,

(6) $$P_{i}≅k_{i}\left(1+{\left|\Gamma_{M}\right|}^{2}\right)P_{IM},$$

where $k_{i}$ is the power dissipation coefficient of TIS. The net absorbed power by the thermistor mount is

(7) $$P_{rf}=\left(1-{\left|\Gamma_{M}\right|}^{2}\right)P_{IM}$$

Therefore, it can be obtained from Equations (5)–(7) that

(8) $$g=1+ck_{i}\frac{1+{\left|\Gamma_{M}\right|}^{2}}{1-{\left|\Gamma_{M}\right|}^{2}}$$

As c and $k_{i}$ are determined by the physical structure and the material property of a waveguide TIS, their product $ck_{i}$ is actually a system constant of the microcalorimeter and denoted as φ in this study. From Equation (8), note that once the system constant $\varphi =ck_{i}$ is obtained, the correction factor g of microcalorimeter for calibrating a thermistor mount with known input reflection coefficient $\Gamma_{M}$ can be determined, and thereby the effective efficiency $\eta_{e}$ of the thermistor mount.

## 3. Determination of the Correction Factor

**Figure 2:**
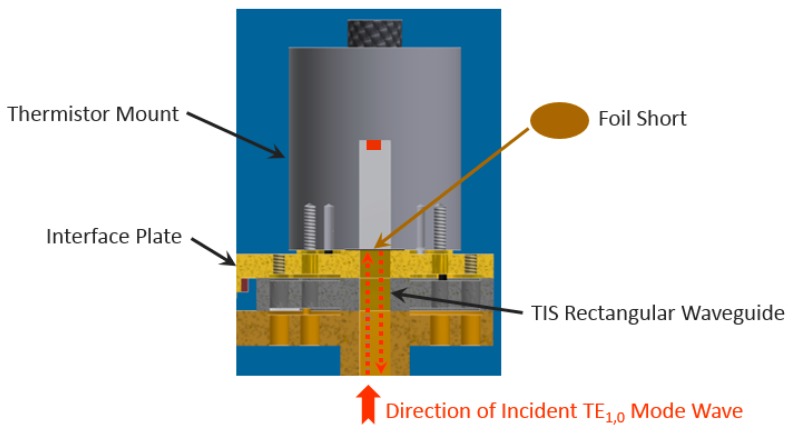
Concept of “foil short” measurements for determining the correction factor of a waveguide microcalorimeter.

“Foil Short” measurement has been well-accepted for experimental determination of the correction factor g [7,11]. A schematic illustration of “Foil Short” measurement is shown in Figure 2 as a reference, where a foil short is inserted between the DUT (thermistor mount) to be calibrated and the interface plate. During the “Foil Short” measurements, the DUT is dc-biased through the Type-IV power meter in a steady status.

With the RF input on, the power $P_{FS}$ dissipated at the foil short and the power $P_{i,FS}$ dissipated at the TIS cause the output voltage change $\Delta e_{FS}$ of the thermopile. Similar to Equation (3), the following relationship can be arrived at

(9) $$\Delta e_{FS}=k\left(P_{FS}+cP_{i,FS}\right).$$

For the foil short with a reflection coefficient of $\Gamma_{FS}$ and incident power of $P_{IFS}$ to the TIS, similar to Equation (6), the dissipated power $P_{i,FS}$ at the TIS can be determined as

(10) $$P_{i,FS}≅k_{i}\left(1+{\left|\Gamma_{FS}\right|}^{2}\right)P_{IFS}.$$

Combining Equations (9) and (10), it can be obtained that

(11) $$\varphi =ck_{i}=\frac{\Delta e_{FS}}{k\left(1+{\left|\Gamma_{FS}\right|}^{2}\right)P_{IFS}}-\frac{P_{FS}}{\left(1+{\left|\Gamma_{FS}\right|}^{2}\right)P_{IFS}}.$$

Since $P_{FS}=P_{IFS}\left(1-{\left|\Gamma_{FS}\right|}^{2}\right)$, conventionally combing (8) and (11), the correction factor g of a microcalorimeter can be determined as

(12) $$g=1+\frac{\Delta e_{FS}}{k\left(1+{\left|\Gamma_{FS}\right|}^{2}\right)P_{IFS}}\times \frac{1+{\left|\Gamma_{M}\right|}^{2}}{1-{\left|\Gamma_{M}\right|}^{2}}-\underset{︸}{\frac{1-{\left|\Gamma_{FS}\right|}^{2}}{1+{\left|\Gamma_{FS}\right|}^{2}}\times \frac{1+{\left|\Gamma_{M}\right|}^{2}}{1-{\left|\Gamma_{M}\right|}^{2}}}.$$

This relationship has been reported in [7,11,12]. However, a recent bilateral comparison [14] of scattering parameter magnitude measurements of WR-15 (50–75 GHz) and WR-10 (75–110 GHz) waveguide type between the Istituto Nazionale di Ricerca Metrologica (INRIM), Italy and the National Metrology Center, A*STAR (NMC), Singapore showed that the uncertainty of reflection coefficient for a “Short” traveling standard can vary from 0.005 to 0.02 (at a 95% confidence level). Higher uncertainty of the reflection coefficient $\Gamma_{FS}$ for foil short can then be propagated to the estimated correction factor g, and thereby the determined effective efficiency $\eta_{e}$. Therefore, it motivates us to find an alternative solution for determining the correction factor g as discussed below.

Through reorganizing (11), we can achieve that

(13) $$\varphi =\frac{\Delta e_{FS}}{k\left(1+{\left|\Gamma_{FS}\right|}^{2}\right)P_{IFS}}\left(1-\frac{kP_{FS}}{\Delta e_{FS}}\right).$$

With (9), it is found that

(14) $$\varphi =\frac{\Delta e_{FS}}{k\left(1+{\left|\Gamma_{FS}\right|}^{2}\right)P_{IFS}}\left(\frac{c}{\frac{P_{FS}}{P_{i,FS}}+c}\right).$$

In this study, a power ratio ρ between $P_{FS}$ and $P_{i,FS}$ is defined as $\rho =P_{FS}/P_{i,\;FS}$, then we can get

(15) $$\varphi =\frac{c}{c+\rho }\times \frac{\Delta e_{FS}}{k\left(1+{\left|\Gamma_{FS}\right|}^{2}\right)P_{IFS}}.$$

Combining (15) with (8), a new correction factor g is proposed in this study for evaluating the waveguide microcalorimeter as follows,

(16) $$g=1+\frac{c}{c+\rho }\times \frac{\Delta e_{FS}}{k\left(1+{\left|\Gamma_{FS}\right|}^{2}\right)P_{IFS}}\times \frac{1+{\left|\Gamma_{M}\right|}^{2}}{1-{\left|\Gamma_{M}\right|}^{2}}.$$

Note that the significant uncertainty portion involving $\Gamma_{FS}$ and $\Gamma_{M}$ as underbraced in Equation (12) has been eliminated in Equation (16); however, with the introduction of another factor determined by power ratio ρ and equivalence factor c which may be under control better. This proposed solution in (16) theoretically may offer a smaller combined uncertainty. The equivalence factor c that considers the thermal paths which are different comparing to those from the thermistor mount to the thermopile approximates to be 0.5 (as representative of all the microcalorimeters in [7]). This is because the relative heating effectiveness through the TIS changes linearly from a value of approximately one at the mount flange to almost zero at the far end as discussed in [7]. As a result, only half of the heating in the TIS is measured by the thermopile.

Therefore, proper determination of the power ratio ρ between $P_{FS}$ (the power dissipated at the foil short) and $P_{i,FS}$ (the power dissipated at the TIS) in “Foil Short” measurements becomes very important for evaluating the system constant φ of a waveguide microcalorimeter, and thereby its correction factor g for calibrating the thermistor mounts. In the following section, we propose to apply the electromagnetic field theory analysis to determine this power ratio theoretically in this study.

## 4. Mathematical Modeling Through Electromagnetic Field Theory Analysis

The properties of waveguides in support of wave propagation and mode are characterized by the presence of longitudinal magnetic or electric field components, and can be derived by electromagnetic field theory analysis ([15] Chapter 3).

**Figure 3:**
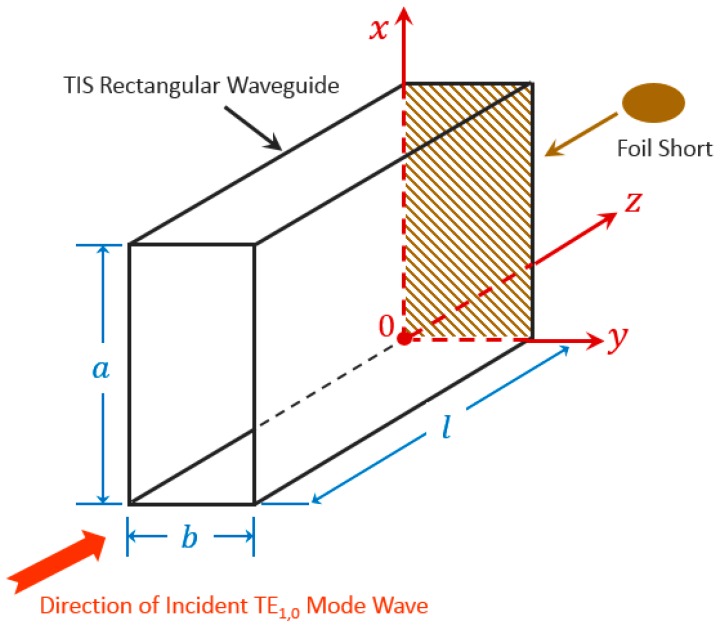
Dimensional illustration of thermal isolation section (TIS) with incident TE_1,0_ mode wave in “Foil Short” measurement.

In a rectangular waveguide, the dominant wave propagating inside is the TE_1,0_ mode. In the following analysis, it is assumed that that both the waveguide walls and the foil short have high conductivity σ and small skin depth δ resulting in small losses (almost lossless, with attenuation constant α ≈ 0), which do not appreciably perturb the TE_1,0_ mode fields. For the incident wave in + *z* direction with a peak amplitude level of *A*, and with a foil short at *z* = 0 along the rectangular waveguide (a>b) as shown in Figure 3, the transverse field components are

(17) $$\{\begin{array}{c} H_{z}=A\left\{\cos\left(\frac{\pi x}{a}\right)\right\}\left(1+\Gamma_{z}e^{j2\beta z}\right)e^{-j\beta z},\\ H_{x}=j\frac{\beta }{K_{c}}A\left\{\sin\left(\frac{\pi x}{a}\right)\right\}\left(1-\Gamma_{z}e^{j2\beta z}\right)e^{-j\beta z},\\ E_{y}=-j\frac{\beta }{K_{c}}Z_{h}A\left\{\sin\left(\frac{\pi x}{a}\right)\right\}\left(1+\Gamma_{z}e^{j2\beta z}\right)e^{-j\beta z},\\ E_{x}=E_{z}=H_{y}=0, \end{array}\;,$$

where $K_{c}$ is the cutoff wave number, β is the phase constant, and $Z_{h}$ is the wave impedance, as follows,

$$K_{c}=\frac{\pi }{a},\;\beta =\frac{2\pi }{\lambda_{g}},and\;Z_{h}=\;\eta \frac{\lambda_{g}}{\lambda }.$$

Here, $\lambda_{g}$ is the guide wavelength and equal to,

$$\lambda_{g}=\frac{\lambda }{\sqrt{1-{\left(\frac{\lambda }{2a}\right)}^{2}}}$$

for the wavelength λ in TE_1,0_ mode. $\Gamma_{z}$ is the voltage reflection coefficient at *z* = 0 (approximately 1 for the foil short) as shown in Figure 3. The incident power $P_{IFS}$ (at *z* = 0) is

(18) PIFS=12Re∫0x=a∫0y=bE→×H→∗·z^dxdy=12(βKc)2ZhA2ab2.

For convenient in calculation, Equation (17) can be reformatted as [16]

(19) $$\{\begin{array}{c} H_{z}=-2jA\cos\left(\frac{\pi x}{a}\right)\sin\beta z,\\ H_{x}=2j\frac{\beta }{K_{c}}A\sin\left(\frac{\pi x}{a}\right)\cos\beta z,\;\\ E_{y}=-2\frac{\beta }{K_{c}}Z_{h}A\sin\left(\frac{\pi x}{a}\right)\sin\beta z. \end{array}\;$$

Note that the dissipated power $P_{s}$ at a wall surface with surface resistance $R_{s}$ is

(20) $$P_{s}=\frac{R_{s}}{2}\iint {\vec{J}}_{s}\times {\vec{J}}_{s}{}^{*}dA,$$

where the surface current density ${\vec{J}}_{s}$ is given by

(21) $${\vec{J}}_{s}≅\hat{n}\times {\vec{H}}_{surface}.$$

Therefore, for the broad wall (a by l) as shown in Figure 3, the magnitudes of the x and z component of the current densities are

(22) $$\left|J_{x}\right|≅2A\cos\left(\frac{\pi x}{a}\right)\sin\beta z,$$

(23) $$\left|J_{z}\right|≅2\left(\frac{\beta }{K_{c}}\right)A\sin\left(\frac{\pi x}{a}\right)\cos\beta z.$$

The magnitudes of the current density in the narrow wall (b by l) is

(24) $$\left|J_{NW}\right|≅2A\sin\beta z.$$

The magnitudes of the current density in the foil short (a by b) is

(25) $$\left|J_{FS}\right|≅2\left(\frac{\beta }{K_{c}}\right)A\sin\left(\frac{\pi x}{a}\right).$$

According to (20), the power $P_{BW}$ dissipated in the two broad walls is

(26) $$P_{BW}=2\cdot \frac{R_{S}}{2}\int_{0}^{x=a}\int_{0}^{z=l}\left({\left|J_{x}\right|}^{2}+{\left|J_{z}\right|}^{2}\right)dxdz≅A^{2}\frac{al}{\sigma \delta }\left\{1+{\left(\frac{\beta }{K_{c}}\right)}^{2}\right\}\;.$$

Similarly, the power $P_{NW}$ dissipated in the two narrow walls is

(27) $$P_{NW}=2\cdot \frac{R_{S}}{2}\int_{0}^{y=b}\int_{0}^{z=l}{\left|J_{NW}\right|}^{2}dydz=4A^{2}\frac{bl}{\sigma \delta }\left\{\frac{1}{2}-\frac{\sin2\beta l}{\beta l}\right\}≅2A^{2}\frac{bl}{\sigma \delta },\;$$

and the power $P_{FS}$ dissipated in the foil short is

(28) $$P_{FS}=\frac{R_{S}}{2}\int_{0}^{x=a}\int_{0}^{y=b}{\left|J_{FS}\right|}^{2}dxdy\;={\left(\frac{\beta }{K_{c}}\right)}^{2}A^{2}\frac{ab}{\sigma \delta }\;.$$

Therefore,

(29) $$\rho =\frac{P_{FS}}{P_{i,\;FS}}=\frac{P_{FS}}{P_{BW}+P_{NW}}=\frac{{\left(\frac{\beta }{K_{c}}\right)}^{2}ab}{2bl+al\left\{1+{\left(\frac{\beta }{K_{c}}\right)}^{2}\right\}}=\frac{{\left(\frac{2a}{\lambda_{g}}\right)}^{2}ab}{2bl+al\left\{1+{\left(\frac{2a}{\lambda_{g}}\right)}^{2}\right\}}.\;$$

Here, it needs to be highlighted that final expression in (29) is achieved with the elimination of the surface resistance $R_{s}$ at both the denominator and numerator. This elimination/simplification is valid only under the assumption that the TIS and the foil short share the same (or approximately the same) electrical characteristics such as conductivity σ and skin depth δ, and if the metal thickness is higher than the skin depth for both the TIS and the foil short.

In practice, these requirements could be achieved during the fabrication of TIS and foil short using the same metal material with enough thickness and with same surface treatment. Together with Equation (16), the correction factor g can be determined properly using (29). In the next section, its performance will be compared with conventional method with detailed discussion.

## 5. Performance Evaluation and Analysis

**Figure 4:**
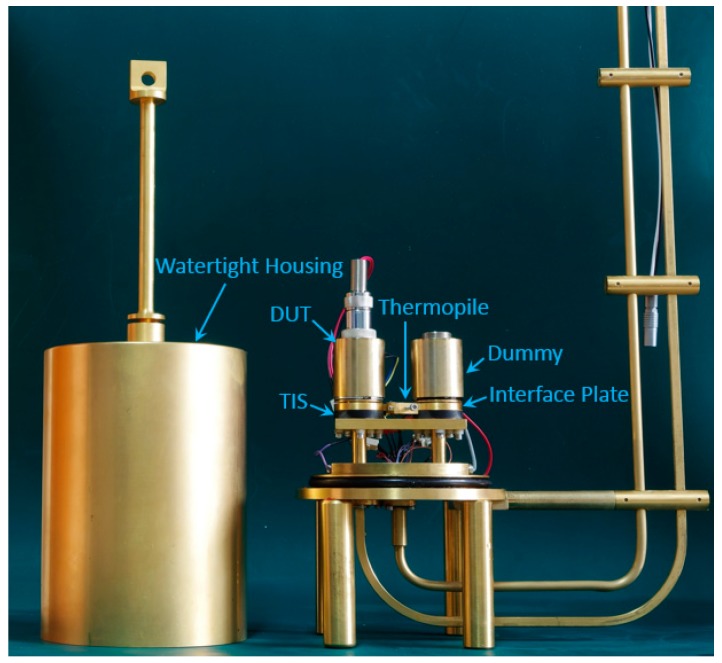
Picture of a WR-15 (50–75 GHz) microcalorimeter assembled at the National Institute of Metrology, China.

Performance of the newly proposed correction factor g in (16) has been evaluated with a WR-15 microcalorimeter that is now serving as the national waveguide primary power standard of China. Figure 4 below shows an assembled WR-15 microcalorimeter that will be used in the evaluation, which covers the frequency range of 50 to 75 GHz.

### 5.1. Performance Evaluation of the Proposed Correction Factor g

The TIS of the fabricated microcalorimeter has dimensions of a = 0.00376 m, b = 0.00188 m, and l = 0.0105 m. Note that the interface plate as shown in Figure 4 has an exactly same size as the TIS.

**Figure 5:**
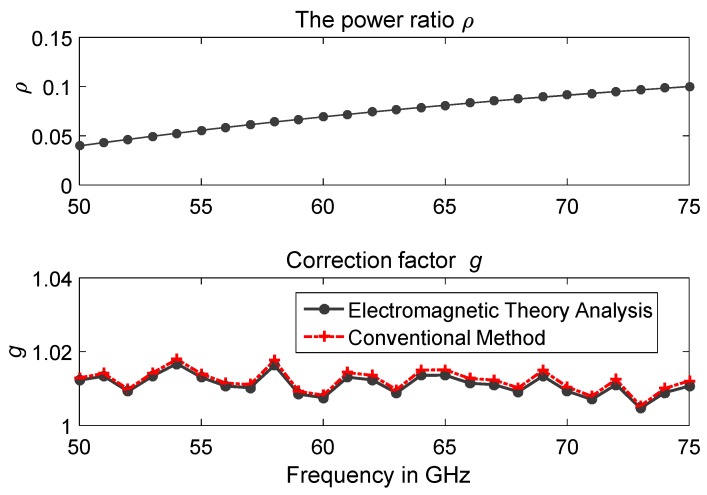
Results of power ratio *ρ*, and comparison of correction factor g for the WR-15 microcalorimeter using the conventional method and the proposed method with electromagnetic field theory analysis.

Figure 5 presents the experimental results of *ρ* and the estimated correction factor g covering the whole frequency band (50–75 GHz) for the fabricated WR-15 microcalorimeter shown in Figure 4, using the proposed method (Equation (16)) and the conventional method (Equation (12)). From Figure 5, good agreement exists between the results for correction factor g from the electromagnetic theory analysis and from the conventional method. The proposed method with electromagnetic theory analysis is then used for evaluating the WR-15 microcalorimeter at the National Institute of Metrology, China. Detailed analysis with uncertainty evaluation are discussed below.

### 5.2. Uncertainty Evaluation

Table 1 shows one example for evaluating the measurement uncertainty of the correction factor *g* of a reference standard (Hughes 45774H-1100 thermistor power sensor), using the proposed method (Equation (16)) at 72 GHz. $P_{1}$ and $e_{1}$ are the DC-biased power and the output voltage of thermopile without RF input respectively, and they are used to experimentally determine k, the proportionality constant that depends on the fraction of power flowing through the thermopile and the thermopile sensitivity [11]. The calculated combined standard uncertainty for correction factor g at 72 GHz is around 0.002, following the “Guide to the Expression of Uncertainty in Measurement” (GUM) [17].

To obtain the measurement uncertainty $\Delta \eta_{e}$ for the effective efficiency $\eta_{e}$ ($\eta_{e}=g\cdot \eta_{e,\;uncor}$) of the thermistor mount under test, it can arrive that

(30) $$\Delta \eta_{e}=\sqrt{{\left(\frac{\partial \eta_{e}}{\partial g}\right)}^{2}\Delta g^{2}+{\left(\frac{\partial \eta_{e}}{\partial \eta_{e,\;uncor}}\right)}^{2}\Delta \eta_{e,\;uncor}^{2}}.$$

The uncertainty for the uncorrected effective efficiency $\eta_{e,uncor}$ was evaluated in the conventional way, and is found to be 0.0013 at 72 GHz. Using Equation (30), the combined standard uncertainty for the effective efficiency of the thermistor mount under test at 72 GHz is 0.0024. That is, the expanded uncertainty is approximately 0.0048 at a level of confidence of approximately 95% assuming a Gaussian distribution.

### 5.3. International Comparison

To further evaluate the performance of the proposed method (Equation (16)) with the newly fabricated WR-15 microcalorimeter shown in Figure 4, an informal international comparison [18] of WR-15 (50 to 75 GHz) power measurements has been arranged among at the National Institute of Metrology (NIM) of China, the National Institute of Standards and Technology (NIST) of USA, the Physikalisch-Technische Bundesanstalt (PTB) of Germany, and the National Metrology Centre (NMC) of Singapore.

**Figure 6:**
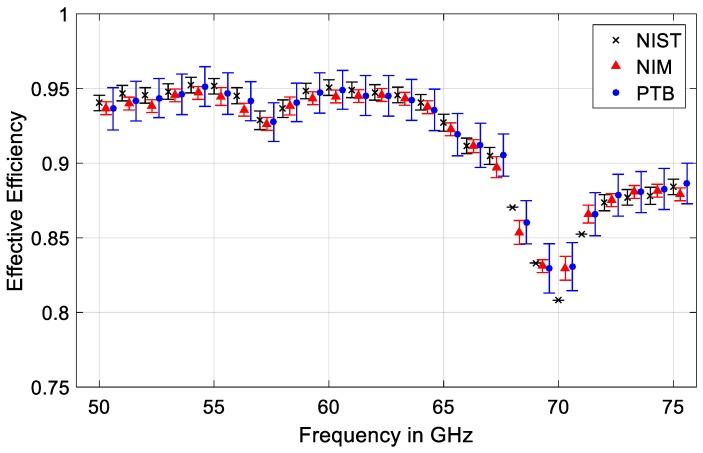
Measured results of the effective efficiency η_e_ for a Hughes 45774H-1100 thermistor power sensor at different laboratories for performance comparison. Uncertainty bars are shown at a level of confidence of approximately 95% assuming a Gaussian distribution. Frequency values are shifted for better readability and no uncertainty value provided by NIST for 68, 69, 70, and 71 GHz.

Comparison results of the measured effective efficiency for one of the traveling standards, Hughes 45774H-1100 thermistor power sensors are presented in Figure 6 for the whole frequency range of 50 to 75 GHz, using the proposed method in this paper and also compared to the primary power measurement systems at NIST and PTB. From the results, good equivalence of power measurements in WR-15 waveguide has been clearly observed among the participating laboratories. This further validates the proposed method and newly developed primary power measurement system at the NIM, China, for calibrating the waveguide RF power sensors.

## 6. Conclusions

In this paper, a new method for determining the correction factor g of a waveguide microcalorimeter was reported, using the electromagnetic field theory to analysis the effect of waveguide TIS in “foil short” measurement mode. The proposed method determines the contribution of the power dissipated within the TIS into the correction factor g, in term of the physical dimensions of the TIS.

The proposed method has been implemented to evaluate a newly fabricated WR-15 microcalorimeter at the NIM, China. The estimated correction factor g of the microcalorimeter using the proposed method has been compared against the conventional method, and good agreements have been observed. To further evaluate its performance, the proposed method with the newly fabricated WR-15 microcalorimeter has been evaluated in an informal international comparison of WR-15 (50 to 75 GHz) power measurements with the NIST of USA, the PTB of Germany and the NMC of Singapore, where good equivalence has been observed.

## Figures and Tables

**Table 1:** Uncertainty budget for correction factor at 72 GHz.

Quantity	Uncertainty Components	Type	Probability Distribution	Standard Uncertainty *u_i_* (*x*)	Sensitivity Coefficient *c*_i_	*u_i_* (*y*) = *c_i_ u_i_* (*x*)
ρ	Caliper	B	Rectangular	0.0005	−0.01371	−0.000007
*P*_1_	Digital multimeter	B	Rectangular	0.001	0.00070	0.000001
$e_{1}$	Digital voltmeter	B	Rectangular	0.04	−0.00002	−0.000001
$\Delta e_{FS}$	Digital voltmeter	B	Rectangular	0.08	0.01520	0.001216
$\Gamma_{FS}$	Network analyzer	B	Rectangular	0.01	−0.01502	−0.000150
$P_{IFS}$	Type IV power meter & Digital voltmeter	B	Rectangular	0.08	−0.01683	−0.001347
$\Gamma_{M}$	Network analyzer	B	Rectangular	0.01	0.00777	0.000078
*g*	Repeatability (Typical)	A	Normal	0.001	1	0.001
Combined Standard Uncertainty for Correction Factor g at 72 GHz						0.002
